# Missed Venous Thromboembolism Prophylaxis in ICU Patients: A Retrospective Cohort Study Using the Medical Information Mart for Intensive Care IV (MIMIC-IV)

**DOI:** 10.7759/cureus.86370

**Published:** 2025-06-19

**Authors:** Rohan Muchintala, Anoshia Khan, Kartik Kalia, Shabaaz M Baig

**Affiliations:** 1 Anesthesiology and Perioperative Medicine, Rutgers University New Jersey Medical School, Newark, USA

**Keywords:** critical care mortality, enoxaparin outcomes, heparin in icu, hospital length of stay, icu quality improvement, in-hospital mortality, missed vte prophylaxis, pharmacologic anticoagulation, retrospective cohort study, vte prevention protocol

## Abstract

Introduction: Venous thromboembolism (VTE), including deep vein thrombosis and pulmonary embolism, is a preventable complication that significantly contributes to morbidity and mortality in intensive care unit (ICU) patients. Despite established guidelines recommending routine prophylaxis, many ICU patients do not receive the indicated doses.

Methods: We conducted a retrospective cohort study using the Medical Information Mart for Intensive Care IV (MIMIC-IV), a publicly available critical care database. Adult patients with a single ICU admission were stratified by receipt of pharmacologic VTE prophylaxis (subcutaneous heparin or enoxaparin). Primary outcomes were ICU length of stay (LOS) and in-hospital mortality. We performed t-tests, chi-square tests, and multivariable logistic regression, adjusting for age, gender, and emergency admission status.

Results: Among 65,355 ICU patients, 85.6% received pharmacologic prophylaxis. Patients who missed prophylaxis had significantly shorter ICU LOS (1.80 vs. 3.80 days, p < 0.001) but higher in-hospital mortality (17.1% vs. 11.6%, p < 0.001). Prophylaxis was associated with a 65% reduction in the adjusted odds of in-hospital death (OR: 0.35; 95% CI: 0.34-0.37).

Conclusions: Missed VTE prophylaxis in ICU patients was associated with an increased in-hospital mortality. These findings support ICU quality improvement initiatives aimed at standardizing prophylaxis protocols to reduce preventable harm.

## Introduction

Venous thromboembolism (VTE), which includes deep vein thrombosis (DVT) and pulmonary embolism (PE), remains one of the most preventable causes of morbidity and mortality among hospitalized patients, particularly those admitted to the intensive care unit (ICU) [1,2]. Critically ill patients are uniquely predisposed to thrombotic events due to a combination of factors, including prolonged immobility, sedation, systemic inflammation, vasopressor use, invasive procedures, and mechanical ventilation [1]. These pathophysiologic features create a hypercoagulable state that warrants prompt and consistent prophylactic intervention.

To address this evidence gap, we conducted a large retrospective cohort study using the Medical Information Mart for Intensive Care IV (MIMIC-IV) database [3]. MIMIC-IV offers a unique opportunity to examine ICU practices and outcomes in a highly granular and standardized format.

National and international societies, including the American College of Chest Physicians (ACCP), Society of Critical Care Medicine (SCCM), and American Society of Hematology (ASH), recommend the routine use of pharmacologic VTE prophylaxis in ICU patients unless contraindicated by active bleeding, severe thrombocytopenia, or significant coagulopathy [1,4,5]. The most commonly used pharmacologic agents in these settings are subcutaneous unfractionated heparin and low-molecular-weight heparin (LMWH), such as enoxaparin. Despite strong evidence supporting the efficacy and safety of prophylaxis, real-world implementation remains inconsistent.

A substantial body of literature has highlighted gaps in prophylaxis delivery across hospital systems. Studies have shown that missed or delayed doses are common, particularly in critically ill patients with dynamic clinical courses [6-9]. Contributing factors include oversight during medication reconciliation, physician discretion in borderline contraindications, nursing documentation errors, and the absence of automated electronic alerts. These lapses are not trivial; evidence suggests that even short interruptions in prophylactic regimens can increase the risk of VTE and subsequent complications, including death [10].

Missed doses of VTE prophylaxis not only pose a direct threat to patient outcomes but also represent a critical target for hospital-based quality improvement (QI) efforts. The Institute for Healthcare Improvement and the Centers for Medicare & Medicaid Services (CMS) have highlighted VTE as a key hospital-acquired condition, underscoring its relevance to public reporting and reimbursement models [11]. Moreover, performance on VTE prophylaxis is often included in institutional quality dashboards and ICU scorecards, linking adherence to broader hospital safety and accreditation standards.

Although the importance of VTE prevention is well-recognized, few large-scale studies have directly quantified the outcome differences between ICU patients who receive prophylaxis and those who do not, particularly in real-world datasets. Prior studies are often limited by small sample sizes, single-center designs, or heterogeneous inclusion criteria, which make it difficult to draw broad conclusions. Additionally, while some randomized controlled trials have assessed the efficacy of prophylaxis in surgical patients, their applicability to the medical ICU population remains uncertain due to differences in patient acuity and baseline thrombosis risk. Therefore, the objective of this study was to evaluate the association between missed pharmacologic VTE prophylaxis and clinical outcomes in ICU patients using the MIMIC-IV database. Specifically, our primary aim was to determine whether missed prophylaxis was associated with an increased in-hospital mortality. Our secondary aim was to compare ICU length of stay (LOS) between patients who received and did not receive prophylaxis.

## Materials and methods

Study design and data source

We conducted a retrospective cohort study using the MIMIC-IV, version 3.1, database [1], a publicly accessible, de-identified clinical dataset developed by the Massachusetts Institute of Technology (MIT) Laboratory for Computational Physiology. MIMIC-IV includes detailed records from over 70,000 ICU admissions at Beth Israel Deaconess Medical Center between 2008 and 2019, covering patient demographics, hospital course, laboratory values, medication administration, diagnoses, and outcomes. The use of MIMIC-IV is approved under the Health Insurance Portability and Accountability Act (HIPAA) Safe Harbor provision. Access to the database was obtained after the completion of required training in human subjects research through the Collaborative Institutional Training Initiative (CITI) program.

Ethical approval

This study was exempt from the institutional review board (IRB) approval because it involved publicly available, de-identified data. All data used were fully anonymized before analysis in accordance with the HIPAA guidelines. All study procedures followed the ethical standards outlined in the Declaration of Helsinki and were consistent with the guidelines of the International Committee of Medical Journal Editors (ICMJE).

Study population and cohort selection

We included adult patients aged 18 years and older who were admitted to the ICU and had a single recorded ICU stay during their hospitalization. Patients with multiple ICU admissions were excluded to reduce complexity in outcome attribution and to prevent overlapping records. We further excluded patients with missing or incomplete demographic data, missing outcome variables, or insufficient prescription records, ensuring analytical robustness. No exclusions were made based on comorbid conditions or illness severity scores due to limitations in the availability of standardized severity indices in the dataset.

Exposure definition

The primary exposure was receipt of pharmacologic VTE prophylaxis during the ICU stay. VTE prophylaxis was defined as the administration of subcutaneous unfractionated heparin or LMWH (enoxaparin), identified through medication order entries. Keyword matching was used to extract prescription events from medication administration records. Patients were then categorized into two cohorts: (1) those who received any pharmacologic VTE prophylaxis during their ICU stay (prophylaxis group) and (2) those with no documented evidence of VTE prophylaxis administration (missed group). It is important to note that the presence of a medication order was used as a proxy for administration. While this may not account for missed or refused doses, it provides a consistent and reproducible method within the limits of the structured electronic health record (EHR) data available.

Outcomes

The primary outcomes were in-hospital mortality and ICU LOS. In-hospital mortality was defined as death during the index hospital admission, while ICU LOS was calculated as the total number of days between ICU admission and ICU discharge. A secondary outcome, 30-day readmission, was initially considered using a proxy variable for multiple hospitalizations recorded under the same patient identifier. However, due to insufficient variation in this field, readmission was excluded from analysis. This limitation likely reflects the structure of the MIMIC-IV database rather than a true absence of readmissions.

Covariates

The following covariates were included based on prior literature and relevance to ICU outcomes. Age was treated as a continuous variable. Gender was encoded as a binary variable (male or female). Admission type was categorized as emergency versus non-emergency and was inferred from the admission_type field to reflect illness acuity. Additional clinical covariates such as Sequential Organ Failure Assessment (SOFA) scores, bleeding risk indices, or laboratory values were not included due to high rates of missing data or non-uniform recording across ICU types.

Statistical analysis

All analyses were conducted using Python-based libraries including pandas, scipy, and statsmodels. Continuous variables, such as ICU LOS and age, were analyzed using Welch’s t-tests to account for potential heteroscedasticity and are reported as mean ± standard deviation (SD). Categorical variables, including in-hospital mortality, were compared using Chi-square (χ²) tests and are presented as frequencies and percentages (N (%)).

To assess the independent association between pharmacologic VTE prophylaxis and in-hospital mortality, we constructed a multivariable logistic regression model. The initial model included age, gender, and emergency admission status. However, due to collinearity and resulting model instability, emergency admission was excluded from the final model to improve parsimony. Adjusted odds ratios with 95% confidence intervals were reported. Model diagnostics confirmed acceptable variance inflation factors, convergence, and no evidence of multicollinearity. A two-sided p-value of <0.05 was considered statistically significant for all analyses.

## Results

Cohort characteristics

A total of 65,355 adult ICU patients met the inclusion criteria. Of these, 55,932 (85.6%) received pharmacologic VTE prophylaxis, while 9,423 (14.4%) did not. Patients in the missed prophylaxis group were older (mean age: 63.0 ± 17.1 years) compared to those who received prophylaxis (60.1 ± 16.8 years; t = 26.48, p < 0.001). Emergency admissions were more common among patients who did not receive prophylaxis (8,361/9,423; 88.7%) versus those who did (47,605/55,932; 85.1%; χ² = 79.06, p < 0.001). Female representation was slightly higher in the prophylaxis group (26,231/55,932; 46.9%) compared to the no prophylaxis group (4,252/9,423; 45.2%; χ² = 6.51, p = 0.01). Detailed baseline characteristics and outcomes are presented in Table 1, where continuous variables are summarized as mean ± SD and categorical variables as N (%). A two-sided p-value of <0.05 was considered statistically significant.

**Table 1:** Baseline characteristics and outcomes of ICU patients by VTE prophylaxis status ICU LOS: intensive care unit length of stay; VTE: venous thromboembolism; SD: standard deviation Baseline characteristics and clinical outcomes of ICU patients stratified by VTE prophylaxis status. Continuous variables are presented as mean ± SD, and categorical variables are presented as absolute count with corresponding percentage. A p-value of <0.05 was considered statistically significant

Variable	No prophylaxis (n = 9,423)	VTE prophylaxis (n = 55,932)	Test statistic (t/χ²)	p-value
Mean age (years)	63.0 ± 17.1	60.1 ± 16.8	*t* = 26.48	<0.001
Female (N, %)	4,252 (45.2%)	26,231 (46.9%)	χ² = 6.51	0.01
Emergency admission (N, %)	8,361 (88.7%)	47,605 (85.1%)	χ² = 79.06	<0.001
ICU LOS (mean, days)	1.80 ± 3.07	3.80 ± 5.11	*t* = 65.37	<0.001
In-hospital mortality (N, %)	1,615 (17.1%)	6,468 (11.6%)	χ² = 111.52	<0.001

ICU length of stay

**Figure 1:**
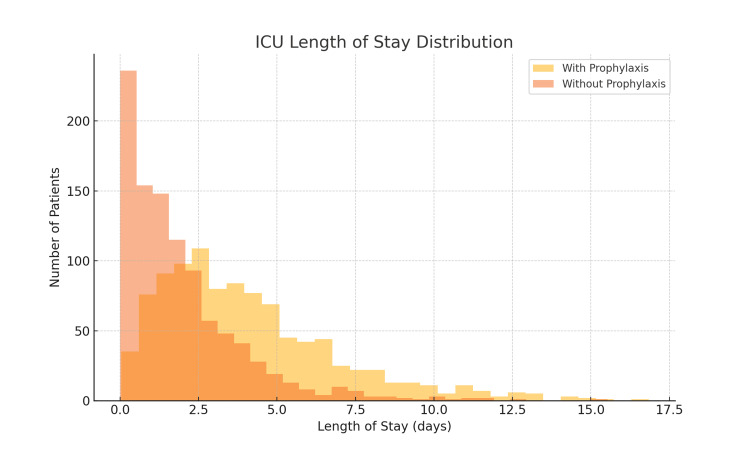
ICU length of stay distribution by VTE prophylaxis status LOS: length of stay; VTE: venous thromboembolism Histogram illustrating the distribution of ICU LOS among patients who received VTE prophylaxis (yellow) versus those who did not (orange). Patients without prophylaxis tended to have shorter ICU stays, while those with prophylaxis showed a broader and longer LOS distribution. Data are presented as frequency counts (N) per LOS interval. A two-sided p-value of <0.05 was considered statistically significant

Patients who received VTE prophylaxis had significantly longer ICU stays (mean: 3.80 ± 5.11 days, n = 55,932) compared to those who did not (mean: 1.80 ± 3.07 days, n = 9,423; t = 65.37, p < 0.001). This counterintuitive finding likely reflects survivorship bias. Patients who died early or experienced rapid clinical deterioration may not have lived long enough to receive consistent prophylaxis or may have had shortened ICU stays due to early death or palliative discharge. The distribution of ICU LOS by prophylaxis status is shown in Figure 1, with data presented as mean ± SD.

In-hospital mortality

In-hospital mortality was significantly higher among patients who did not receive VTE prophylaxis (1,615/9,423; 17.1%) compared to those who did (6,468/55,932; 11.6%; χ² = 111.52, p < 0.001). After adjusting for age, gender, and emergency admission status in a multivariable logistic regression model, receipt of prophylaxis was associated with significantly lower odds of in-hospital death (adjusted OR: 0.35; 95% CI: 0.34-0.37; p < 0.001). Adjusted odds ratios are presented in Table 2.

**Table 2:** Adjusted odds of in-hospital mortality among ICU patients by VTE prophylaxis status and covariates aOR: adjusted odds ratio; CI: confidence interval; VTE: venous thromboembolism Multivariable logistic regression model evaluating the association between VTE prophylaxis and in-hospital mortality, adjusting for age (continuous), gender, and emergency admission status. VTE prophylaxis was associated with significantly reduced odds of mortality. P-values were derived from multivariable logistic regression. A p-value < 0.05 was considered statistically significant

Variable	Adjusted odds ratio (95% CI)	Test statistic (z)	p-value
VTE prophylaxis	0.35 (0.34-0.37)	-44.85	<0.001
Female (vs. male)	0.63 (0.60-0.65)	-21.48	<0.001
Age (per year)	0.99 (0.99-0.99)	-34.75	<0.001

**Figure 2:**
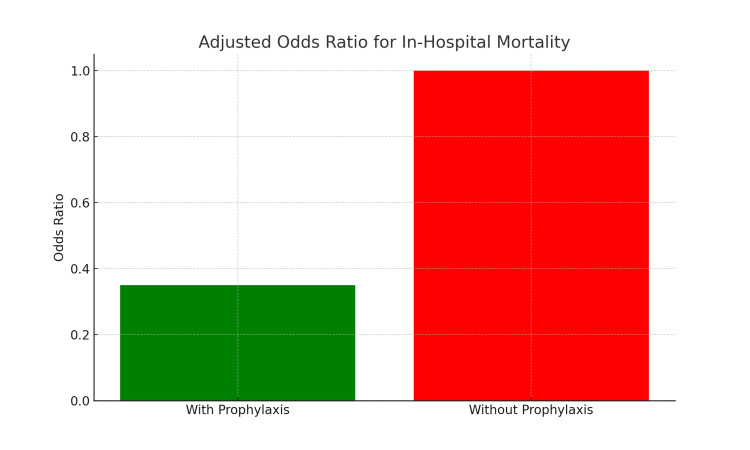
Adjusted odds ratios for in-hospital mortality by VTE prophylaxis and covariates aOR: adjusted odds ratio; CI: confidence interval; VTE: venous thromboembolism Bar chart displaying aORs with 95% confidence intervals from a multivariable logistic regression model evaluating predictors of in-hospital mortality among ICU patients. Variables included in the model were VTE prophylaxis status, age (continuous), gender, and emergency admission status. Receipt of VTE prophylaxis was independently associated with significantly reduced odds of in-hospital mortality. A p-value of <0.05 was considered statistically significant

These adjusted associations are visually represented in Figure 2, which displays the multivariable logistic regression-derived adjusted odds ratios with 95% confidence intervals for in-hospital mortality by VTE prophylaxis status and covariates.

Model diagnostics and sensitivity analysis

The logistic regression model demonstrated good overall fit with no evidence of multicollinearity (all VIFs < 2.0). Residual plots showed no major violations of assumptions. Inclusion of interaction terms between gender and admission type did not improve model performance and was excluded for parsimony.

Sensitivity analyses limited to emergency admissions showed consistent findings, with prophylaxis remaining associated with reduced mortality. Subgroup analysis stratified by age (<65 vs. ≥65 years) also supported the mortality benefit of prophylaxis in both age groups, although absolute mortality rates were higher in older patients.

## Discussion

This large-scale retrospective cohort study of over 65,000 ICU patients provides compelling evidence that missed pharmacologic prophylaxis against VTE is independently associated with increased in-hospital mortality. Despite strong guideline recommendations and the well-documented risks of thrombotic complications in critically ill patients, nearly 15% of patients in our cohort did not receive pharmacologic prophylaxis [1,2,4]. These findings reaffirm the clinical and safety implications of prophylaxis adherence in the intensive care setting [5,11].

Our analysis showed that patients who missed VTE prophylaxis had significantly higher in-hospital mortality (17.1%, n = 1,615) compared to those who received it (11.6%, n = 6,468), even after adjusting for age, gender, and emergency admission status. This association remained robust in sensitivity analyses restricted to emergency admissions and across age strata. While causality cannot be established in a retrospective design, the magnitude and consistency of this effect align with prior findings that highlight the mortality burden of preventable thrombotic events in hospitalized patients [6,12].

Interestingly, we observed a shorter ICU LOS among patients who did not receive prophylaxis. This finding may initially appear counterintuitive but is likely explained by early in-hospital mortality in the missed prophylaxis group. Patients who die early during admission may not survive long enough to receive full VTE prophylaxis or benefit from prolonged ICU management. This survivorship bias has been documented in prior ICU studies evaluating outcomes concerning procedural and pharmacologic interventions [3,13].

Nevertheless, the higher in-hospital mortality in patients who missed prophylaxis (1,615 of 9,423; 17.1%) compared to those who received it (6,468 of 55,932; 11.6%) suggests that survivorship bias alone does not fully account for the observed difference in outcomes. While early death may have limited the opportunity for some patients to receive pharmacologic VTE prophylaxis, the consistently higher mortality rate in the missed group indicates that lack of prophylaxis may contribute directly to worse outcomes. This interpretation is consistent with prior studies demonstrating increased risk of thromboembolic events and associated mortality among critically ill patients who do not receive appropriate prophylaxis [3,13].

From a systems perspective, missed prophylaxis likely reflects workflow inefficiencies, lack of real-time monitoring, or provider hesitancy due to bleeding concerns. Past studies have shown that clinical decision-making in the ICU can be highly variable, especially when prophylaxis is delayed due to active bleeding, procedural timing, or perceived futility [14,15]. However, even short lapses in anticoagulation can result in thrombotic complications, highlighting the need for standardized order sets and automated reminders [16-18].

Our findings have several implications for ICU quality improvement. First, they suggest the urgent need for improved prophylaxis surveillance systems that flag missed doses or delays in initiation. EHR-integrated tools that generate real-time alerts for missed VTE prophylaxis have shown success in reducing omission rates and improving compliance [16,17]. Second, provider education on balancing bleeding risk versus thrombotic risk remains essential. While prophylaxis may be intentionally withheld in patients with coagulopathy or active bleeding, many omissions are unintentional or due to oversight [7,8]. Third, these results support efforts to incorporate VTE prophylaxis into ICU performance metrics and patient safety dashboards. National organizations such as the Agency for Healthcare Research and Quality (AHRQ) and the CMS already monitor VTE prophylaxis as a quality indicator [19]. Institutional benchmarking against expected prophylaxis rates, particularly in academic or high-acuity ICUs, may help identify departments or shifts with suboptimal performance [15].

The study also underscores the value of leveraging large, real-world EHR datasets like MIMIC-IV to examine clinical practice gaps at scale. Unlike single-center chart reviews or small audits, databases like MIMIC allow for high-powered retrospective analysis and support hypothesis-generating research to inform policy and practice [9,10]. Our work contributes to a growing body of literature using open-access EHR data for quality improvement studies in critical care [9,13].

Nevertheless, the internal consistency of these findings, the large sample size, and the use of multivariable modeling lend strength to the observed associations. Although residual confounding is possible, particularly due to the lack of granular clinical variables such as illness severity scores or explicit documentation of contraindications, the robustness of the mortality difference suggests a meaningful clinical signal. These findings underscore the critical importance of reliable, protocol-driven delivery of pharmacologic VTE prophylaxis in the ICU. Future quality improvement efforts should focus on enhancing system-level safeguards, such as automated alerts and standardized protocols, to minimize preventable omissions and improve patient outcomes in critical care environments.

That said, caution is warranted in interpreting these results. The association between missed prophylaxis and mortality is likely influenced by unmeasured confounders. For instance, we were unable to control for illness severity scores (e.g., SOFA, Acute Physiology and Chronic Health Evaluation II (APACHE II)), transfusion data, or specific contraindications to anticoagulation, which may have justified the omission of prophylaxis. Furthermore, medication administration data in MIMIC reflect orders, not confirmed bedside administration, introducing potential misclassification bias [9].

## Conclusions

This large-scale retrospective cohort study highlights a significant association between missed pharmacologic VTE prophylaxis and increased in-hospital mortality among critically ill patients. Despite widespread awareness of VTE risks in the ICU and the availability of effective prophylactic strategies, our findings suggest that lapses in administration remain both prevalent and clinically consequential. These results reinforce the importance of adherence to established guidelines and underscore the potential harms associated with unintentional omission of care.

Beyond observational insight, these findings have meaningful implications for clinical operations. In a resource-intensive environment such as the ICU, protocolized interventions are essential. Our results support the use of structured safeguards, such as default prophylaxis order sets, electronic medication reminders, and daily multidisciplinary checklist reviews, to ensure consistency and accountability in prophylaxis delivery. These system-level strategies have demonstrated success in other domains of critical care and may prove equally effective in reducing missed VTE prophylaxis. Future research should examine the causal pathways contributing to missed prophylaxis, explore risk stratification among ICU subpopulations (e.g., surgical vs. medical, trauma vs. sepsis), and evaluate the impact of targeted quality improvement initiatives on both clinical outcomes and cost-effectiveness. Ultimately, translating these findings into real-world practice may reduce preventable ICU mortality and strengthen the overall safety culture in critical care settings.
